# The influence of tactical positioning on performance in sprint cross-country skiing

**DOI:** 10.1371/journal.pone.0287717

**Published:** 2023-06-23

**Authors:** Pål Haugnes, Jan Kocbach, Dionne Noordhof, Rune Kjøsen Talsnes, Gertjan Ettema, Øyvind Sandbakk

**Affiliations:** 1 Centre for Elite Sports Research, Department of Neuromedicine and Movement Science, Faculty of Medicine and Health Sciences, Norwegian University of Science and Technology, Trondheim, Norway; 2 NORCE Norwegian Research Centre AS, Bergen, Norway; 3 Department of Sports Science and Physical Education, Nord University, Bodø, Norway; Universita degli Studi di Verona, ITALY

## Abstract

The purpose of this study was to examine the influence of tactical positioning on performance in the heats of sprint cross-country (XC) skiing among men and women and the consistency of overtaking events over repeated competitions on the same racecourse. Thirty male and thirty female elite to world-class level skiers within each competition [(sprint International Ski and Snowboard Federation (FIS) points: 40 ± 21 vs. 35 ± 24)] performed two repeated world-cup competitions at four different venues (two in the classical and two in the skating style) between 2017 and 2020. The intermediate rankings at five checkpoints were analysed using television broadcasts of the competitions. Sprint time-trial (STT) rank correlated positively with the final rank for the seven men’s (ρ = .54-.82, *P* < .01) and the eight women’s (ρ = .40-.80, *P* < .05) competitions, while one of the classical competitions for males did not correlate significantly (*P* = .23). The strength of the correlation coefficients between intermediate ranks and final ranks during the heats increased gradually from the first to the last checkpoint among both sexes in the classical style (τ = ~0.26 to ~0.70) and in the skating style (τ = ~0.22 to ~0.82), in which the majority of performance-variance was decided before the start of the finish sprint. For both sexes, ~20 and 16 overtaking events were observed in each heat for the classical and skating style, respectively. There was a significant sex-difference in the number of overtaking events in one out of the 16 competitions (*P* < .01), but no differences across seasons for any competition (*P* = .051–796). Overall, this study showed the importance of tactical positioning for performance in sprint XC skiing, with the number of overtaking events being relatively consistent for competitions performed on the same racecourse.

## Introduction

A sprint cross-country (XC) skiing competition involves a qualifying sprint time-trial (STT), from which the thirty fastest skiers qualify for the subsequent knockout heats (i.e., five quarterfinals [QFs], two semi-finals [SFs], and one final [F]). Here, six skiers in each heat compete against each other for the top-two ranks that qualify for the next round along with the two fastest overall times. The ~3-minute races are performed on varying terrain over a 1.0–1.8 km racecourse, in which the skiers continuously change between sub-techniques (gears) and adapt according to topography and external conditions, such as friction, temperature and wind. The four races within each sprint competition are separated by ~15 to 60-minute breaks, where the ability to recover rapidly is important for performance in the subsequent heats [1–13]. Moderate to large correlations between STT rank and final rank of a sprint competition have been reported [8, 14], and Spencer et al. [8] found a stronger correlation for men than for women, particularly evident in the skating style.

The skiers’ race tactics (including tactical positioning) is regarded particularly important for performance since the final rank in each heat determines whether you advance to the subsequent heat and possibly win the competition. Research on XC skiing mass-start competitions [15] indicate that the positioning of a skier in the pack can both positively and negatively influence energy expenditure and performance. Skiing at the back of the pack may reduce air drag [16] and ski-snow friction [17–19] when compared to skiing at the front. However, this strategy also carries a trade-off, as being too far behind increases the risk of accidents and the accordion effect, which may require more energy for subsequent overtaking events. Although the number of skiers is lower in sprint compared to mass-start events, the same aspects are expected to be of importance during a heat in sprint XC skiing. Here, tactical positioning and overtaking events are crucial, as competitors not only have to determine their own tactical behaviour but must also consider the actions of their opponents. Previously, Andersson et al. [13] found that most heat winners (61%) in a simulated skating competition were leading already after the initial 30 meters of their respective heats. Moreover, it was shown that the strength of the correlations between intermediate rankings at given checkpoints and the final rank ranged between 0.51–0.84, with increased strength towards the finish line. In contrast, Haugnes et al. [14] reported that the best performers in a male classical sprint competition awaited to position themselves at the front of the heat until approaching the final uphill, after which a clear reduction in the number of overtaking events were seen. However, whether these findings differ between the classical and skating style, racecourses, and sexes has not yet been investigated.

Since the period investigated by Spencer et al. [8] and Andersson et al. [13], sprint XC skiing has evolved, for example by more women being specialized in sprint and both sexes more often competing on the same racecourse. Accordingly, there is a need for a better understanding and more updated information related to tactical positioning in sprint XC skiing. In this context, knowledge about the consistency of the number and location of overtaking events, when a competition is repeated on the same racecourse (and comparable conditions) is of interest for understanding the repeatability of “heat behaviour” which also has practical relevance for subsequent racecourse preparations.

Therefore, the purpose of the present study was to examine the influence of tactical positioning on performance in the heats of sprint XC skiing among men and women and the consistency of overtaking events over repeated competitions on the same racecourse.

## Methods

### Design and participants

Thirty male and thirty female XC skiers in each competition, who performed two repeated competitions at four different venues (two in the classical and two in the skating style; in total eight competitions for each sex) between the seasons 2017 and 2020 were analysed. The skiers’ performance level [(sprint International Ski and Snowboard Federation (FIS) points: 40 ± 21 vs. 35 ± 24)] ranged from Tier 4 (elite/international level) to Tier 5 (world-class level) as defined by McKay et al. [20]. Given data are in the public domain, written consent from athletes and ethical approval was not required.

### Measurements

The inclusion criteria for the analysed competitions in this study were that the competitions needed to be held before the 2019/2020 season with the same style, racecourse, and comparable environmental conditions. A total of 15 competitions were assessed for eligibility and 4 competitions were included in the analysis. The competitions at locations A, B, C, and D were performed in the 2017/2018 or 2018/2019 season and then repeated on the same racecourse during the 2019/2020 season. Official competition results were downloaded from the publicly available FIS online database (www.fis-ski.com). Video analysis of tactical positioning and incidents (obstruction, fall, pole break) were obtained by publicly available television broadcast (Norwegian Broadcasting Corporation [NRK] and TV 2 Group). For these analyses, the racecourses were separated into 5 segments (S1-S5) using 5 checkpoints based on terrain topography and classified according to the following criteria; out of stadium (checkpoint 1), crucial parts e.g., major uphill (checkpoint 2 and 3), before the finish sprint (checkpoint 4), and finish (checkpoint 5). Obstruction was classified according to the following classification criteria; skier A suffered loss of speed and/or rank due to skier B performing a non-regulatory track change.

### Statistical analysis

All data are presented as mean ± standard deviation (SD), unless otherwise stated. Shapiro-Wilk test, visual inspection of Q-Q plots, and comparison of histograms were used to assess normality. In cases of non-normally distributed data, a nonparametric alternative was used. The Spearman rank-order correlation coefficient was performed to assess the relationship between the STT rank and the final rank. Tactical positioning for heat winners was explored. During the heats, the percentage of heat winners’ checkpoint positioning (i.e., rank 1–6) was determined. Positioning of all skiers was examined by assessing the relationship between intermediate rankings and final rankings by using Kendall tau-b correlations. A two-way mixed ANOVA was conducted to determine whether there was a statistically significant mean difference in overtaking events between men and women (between group comparison) on the same racecourse between seasons (within subject comparison). Levene’s test was used to assess the homogeneity of variances. The magnitude of the correlation coefficients and effect sizes was interpreted as follows: 0.0–0.1, trivial; 0.1–0.3, small; 0.3–0.5 moderate; 0.5–07, large; 0.7–0.9, very large; 0.9–1.0, nearly perfect [21]. The statistical significance level was set at α<0.05. All statistical analyses were performed using STATA 16.0 software (Stata Corporation, College Station, TX, USA) and Office Excel 2016 (Microsoft Corporation, Redmond, WA, USA).

## Results

### The relationship between STT rank and the overall ranking

There were large positive correlations between STT rank and the final rank for male skiers both in the competitions using the classical (ρ = .65-.82, all *P* < .01) and skating styles (ρ = .54-.82, all *P* < .01), except for the 2019–2020 season in the classical competition A (*P* = .23). For the female skiers, moderate to large positive correlations were found between STT rank and the final rank in the classical style (ρ = .40-.78, *P* < .05) and large positive correlations in the skating style (ρ = .57-.80, all *P* < .01).

### External conditions, time, and positioning

**Fig 1:**
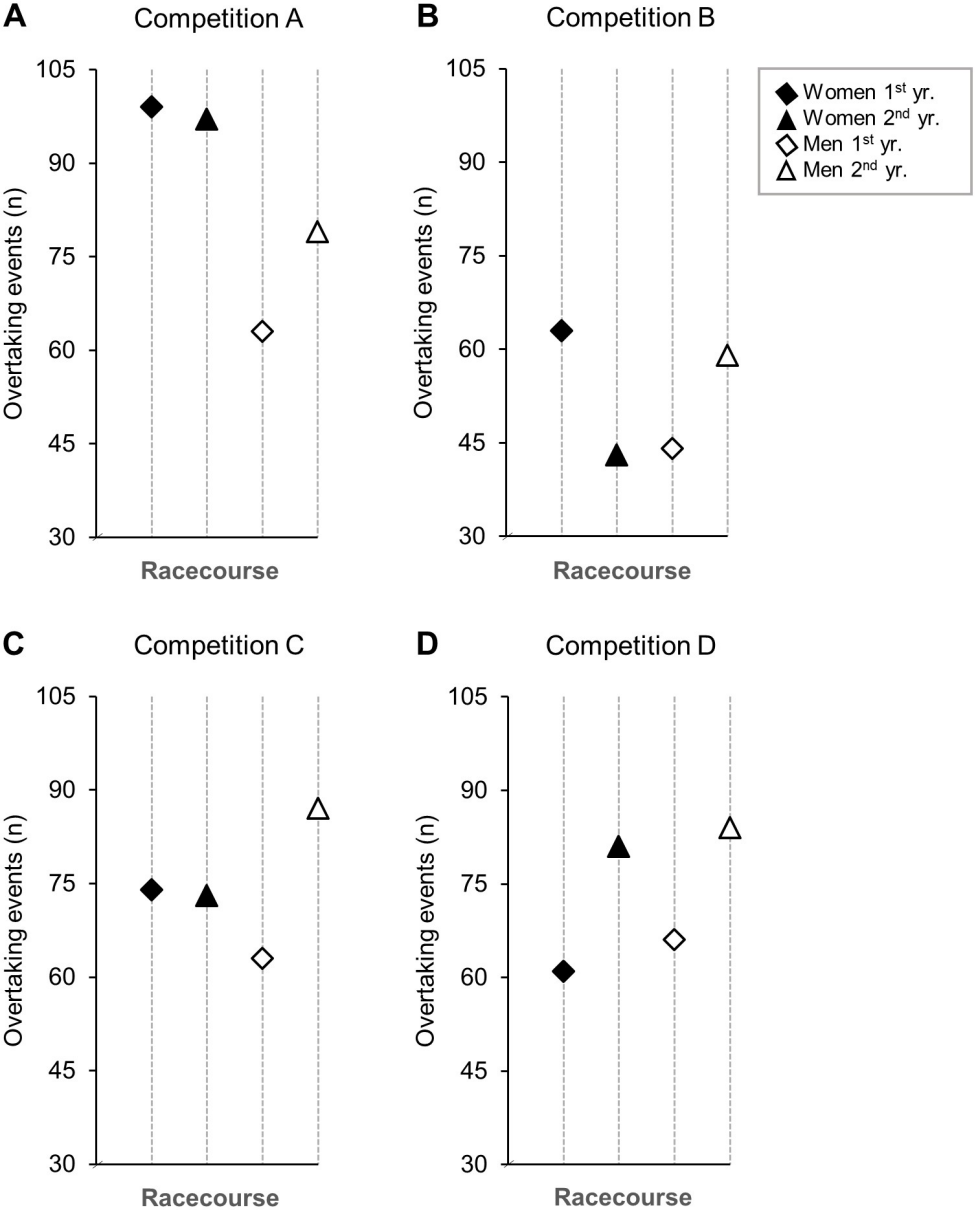
The total number of overtaking events. Overtaking events in two repeated sprint cross-country skiing competitions at four different venues (two classical and two skating style) [(N = 30 male and N = 30 female skiers within each of the eight competitions in this study)].

**Fig 2:**
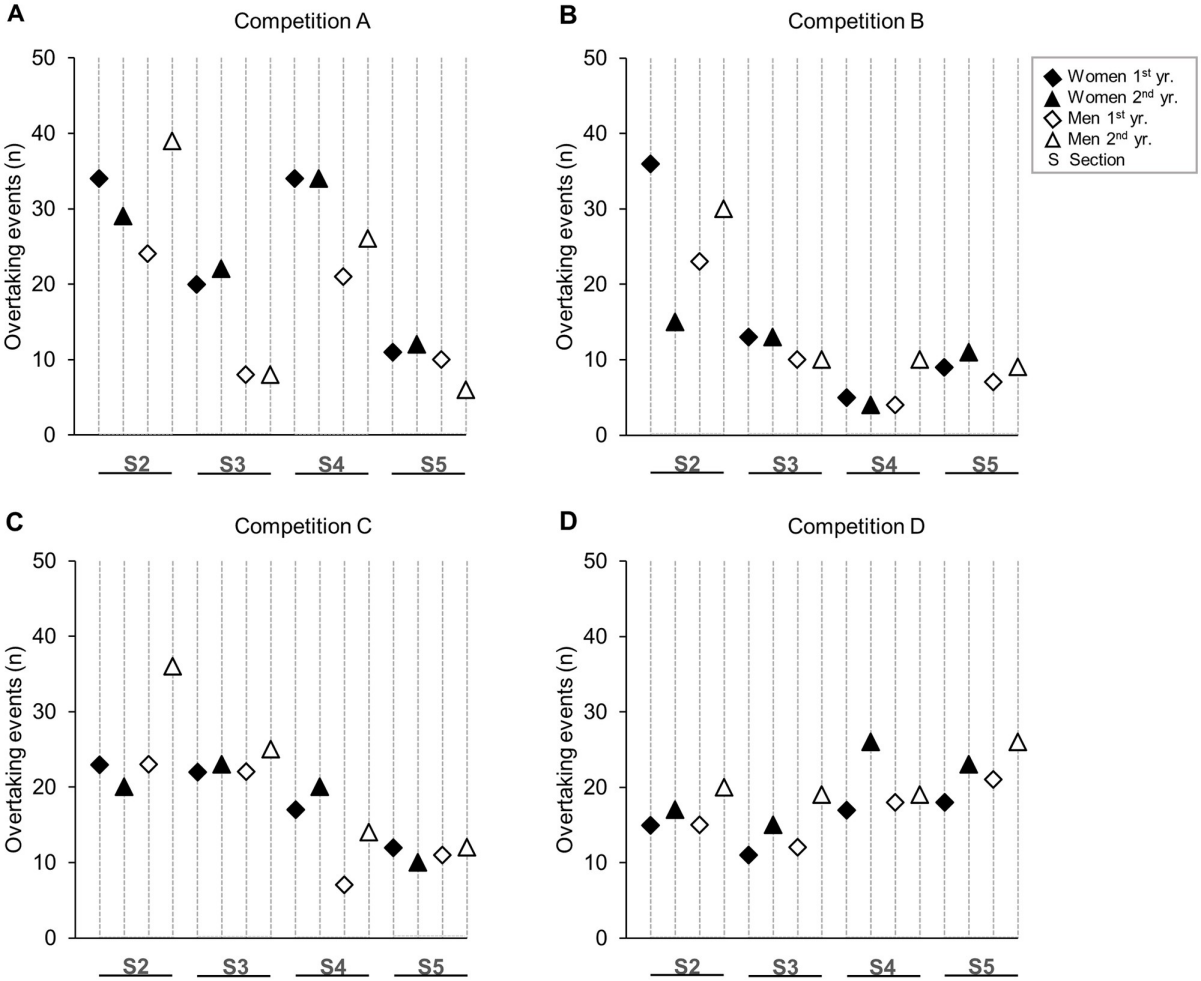
The number of overtaking events in different segments. Overtaking events in two repeated sprint cross-country skiing competitions at four different venues (two classical and two skating style) [(N = 30 male and N = 30 female skiers within each of the eight competitions in this study)].

Overview of topography, weather, snow temperature, performance level, and number of incidents for all competitions analysed in this study are shown in Table 1. The total number of overtaking events for the competitions are shown in Fig 1, and the number of overtaking events on the different segments are shown in Fig 2. For both sexes, an average of 20 and 16 overtaking events were observed in each heat for the classical and skating style, respectively.

**Table 1:** Overview of two repeated sprint cross-country skiing competitions held at four different venues (two classical and two skating). Presented as absolute values [N = 30 male and N = 30 female skiers within each of the eight competitions in this study].

Variable	Competition A classical				Competition B skating				Competition C skating				Competition D classical			
	Women1^st^ yr	Men 1^st^ yr	Women 2^nd^ yr	Men 2^nd^ yr	Women 1^st^ yr	Men 1^st^ yr	Women 2^nd^ yr	Men 2^nd^ yr	Women 1^st^ yr	Men 1^st^ yr	Women 2^nd^ yr	Men 2^nd^ yr	Women 1^st^ yr	Men 1^st^ yr	Women 2^nd^ yr	Men 2^nd^ yr
**FIS (sprint points)**	34 ± 24	42 ± 24	39 ± 26	40 ± 23	31 ± 25	43 ± 21	41 ± 26	37 ± 19	40 ± 25	46 ± 23	43 ± 27	40 ± 19	27 ± 14	38 ± 21	30 ± 18	35 ± 18
**Air temp (°C)**	-0.5	-0.6	-4.0	-4.0	-1.9	-1.9	-2.0	-2.0	0.8	0.8	2.7	2.7	0.7	0.8	0.5	0.0
**Snow temp (°C)**	-2.6	-2.5	-2.6	-2.6	-2.8	-2.8	-1.8	-1.8	0.2	0.3	-2.6	-2.6	-1.4	-1.2	-1.3	-1.3
**Distance (m)**	1400	1400	1400	1400	1500	1500	1500	1500	1500	1500	1500	1500	1214	1214	1200	1200
**HD (m)**	23	23	23	23	18	18	18	18	20	20	25	25	25	25	24	24
**MC (m)**	22	22	22	22	12	12	12	12	16	16	22	22	23	23	21	21
**TC (m)**	43	43	43	43	46	46	46	46	40	40	54	54	36	36	45	45
**Laps (n)**	1	1	1	1	2	2	2	2	2	2	2	2	1	1	1	1
**Pole breaks (n)**	0	1	0	1	1	2	0	1	2	0	1	1	0	0	0	0
**Falls (n)**	1	0	0	0	1	2	2	0	2	0	3	3	1	1	0	2
**Obstructions (n)**	3	3	4	0	1	2	6	4	1	1	1	6	9	4	4	3
**Yellow cards (n)**	4	2	0	2	0	0	1	1	0	0	0	0	0	3	3	0

Abbreviations: HD, height difference; MC, max climb; TC, total climb

There were no significant interactions between sex and seasons on overtaking events in any of the competitions (*P* = .086–0.91). No significant differences in the number of overtaking events were found across seasons for any of the competitions (*P* = .051-.796). However, there was a significant difference in the number of overtaking events between sexes for competition A (*P* = 0.004), but not for competition B-D (*P* = .692-.849). See S1 Appendix for more information.

**Fig 3:**
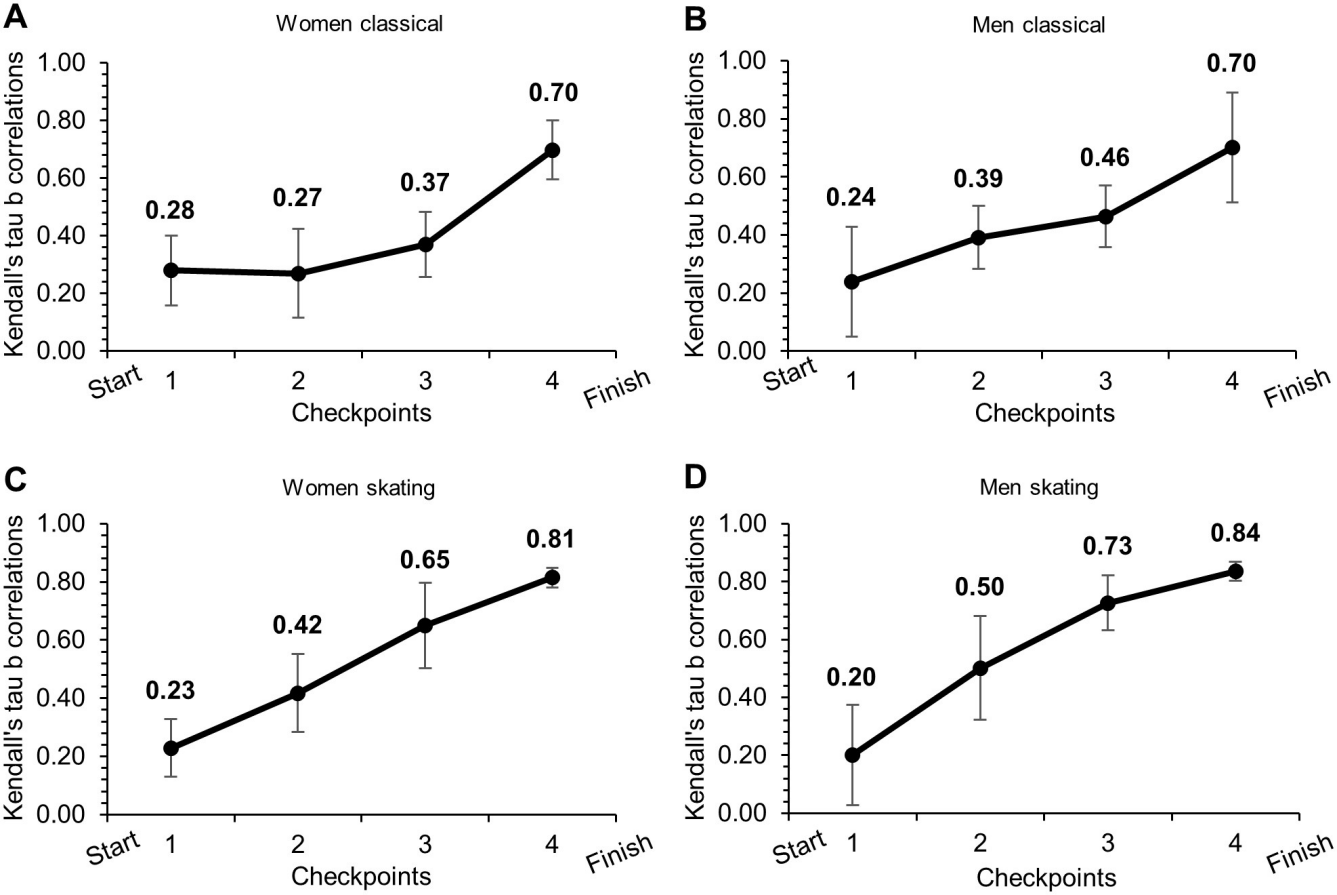
Kendall’s tau-b correlations. The relationship between intermediate ranks at given checkpoints and final rank in two repeated sprint cross-country skiing competitions at four different venues (two classical and two skating style) [N = 30 male and N = 30 female skiers within each of the eight competitions in this study].

The heat winner’s positions are presented in Table 2. Of the heat winners, ~53% of men and ~69% of women in the classical style, and ~63% of men and ~75% of women in the skating style, were positioned first in the heat at the last checkpoint before the start of the finish sprint. Of those ranked among the top-two at the finish, 31–44% were outside the top-two when leaving the stadium (i.e., checkpoint 1), while 34–44% of the heat winners were leading when leaving the stadium. The relationship between intermediate ranks at given checkpoints and final ranks for all skiers during the heats are presented in Fig 3. The average correlation (τ) for men ranged from 0.24 to 0.70 in the classical style and 0.20 to 0.84 in the skating style, whereas the average correlation for women ranged from 0.28 to 0.70 in the classical style and 0.23 to 0.81 in the skating style.

**Table 2:** The average percentage distribution of intermediate rank of the heat winner at various checkpoints is presented for classical (left) and skating (right) styles during two repeated sprint cross-country skiing competitions held at four different venues (two classical and two skating). Presented as percentages [N = 8 male and N = 8 female skiers within each of the eight competitions in this study].

Variable	Heat winner classical						Heat winner skating					
**Checkpoints 1–5 Women**	**Rank 1**	**Rank 2**	**Rank 3**	**Rank 4**	**Rank 5**	**Rank 6**	**Rank 1**	**Rank 2**	**Rank 3**	**Rank 4**	**Rank 5**	**Rank 6**
**1. Out of stadium**	44%	19%	12%	0%	12%	13%	38%	31%	6%	19%	6%	0%
**2. End of first part**	44%	29%	16%	0%	4%	7%	31%	44%	25%	0%	0%	0%
**3. End of middle part**	50%	28%	16%	0%	6%	0%	50%	31%	13%	6%	0%	0%
**4. Before the finish sprint**	69%	25%	6%	0%	0%	0%	75%	25%	0%	0%	0%	0%
**5. Finish**	100%	0%	0%	0%	0%	0%	100%	0%	0%	0%	0%	0%
**Checkpoints 1–5 Men**	**Rank 1**	**Rank 2**	**Rank 3**	**Rank 4**	**Rank 5**	**Rank 6**	**Rank 1**	**Rank 2**	**Rank 3**	**Rank 4**	**Rank 5**	**Rank 6**
**1. Out of stadium**	34%	25%	16%	6%	19%	0%	39%	20%	20%	7%	7%	7%
**2. End of first part**	29%	39%	14%	11%	7%	0%	44%	31%	0%	6%	19%	0%
**3. End of middle part**	44%	34%	6%	9%	7%	0%	50%	44%	6%	0%	0%	0%
**4. Before the finish sprint**	53%	41%	3%	0%	3%	0%	63%	37%	0%	0%	0%	0%
**5. Finish**	100%	0%	0%	0%	0%	0%	100%	0%	0%	0%	0%	0%

Checkpoint 5 Finish: All heat winners were ranked 1.

## Discussion

The purpose of this study was to examine the influence of tactical positioning on performance in the heats of sprint cross-country (XC) skiing among men and women and the consistency of overtaking events over repeated competitions on the same racecourse. The main findings were as follows: 1) the moderate to large correlations between STT rank and final ranks found for most competitions were comparable between styles and sexes; 2) the strength of the correlation coefficients between intermediate ranks and final ranks during the heats gradually increased towards the last checkpoint before the finish sprint for both styles and sexes, with 94% in the classical style and 100% in the skating style being ranked top-two before the finish sprint; 3) for both sexes, ~20 overtaking events in the classical and ~16 overtaking events in the skating style were observed in each heat; 4) the number of overtaking events were consistent i.e., there were no significant differences between seasons for competitions organized on the same type of racecourse under comparable racing conditions.

As previously shown by Spencer et al. [8] and Haugnes et al. [14], moderate to large correlations between STT rank and final rank among both sexes were found for most competitions. However, in contrast to Spencer et al. [8], who found stronger correlations for women than for men in the classical style, such sex-difference was not evident in the present study. This may be explained by a more homogeneous performance level among women in the present study compared to the study of Spencer et al. [8], and that both sexes are now more often competing on the same racecourse. The strength of the correlations shows that a large portion of the variance could not be explained by performance in the STT, meaning that additional factors play a role for performance in the heats such as race tactics and the ability to recover between heats during the competition day. Therefore, future studies should investigate the relationship between recovery and heat performance since the highest ranked skiers in the STT, QFs and SFs could potentially choose subsequent heats with longer or shorter recovery times [22].

Consistent with the findings of Haugnes et al. [14], the correlation coefficients between intermediate ranks and final ranks during the heats showed a gradual increase from the first to the last checkpoint, for both sexes and styles. It was observed that 94% of the heat winners in the classical style and 100% of the heat winners in the skating style were ranked among the top-two at the last checkpoint before the finish sprint. However, it is noteworthy that 31–41% of those ranked among the top-two at the finish were outside the top-two when leaving the stadium (i.e., checkpoint 1), while 34–44% of the heat winners were leading when leaving the stadium. In contrast, Andersson et al. [13], found that most heat winners (61%) of both sexes were in front already after the initial 30 meters of their respective heats, and that 95% of the heat winners were ranked top-two out of the stadium. This difference might be explained by the fact that there was a larger heterogeneity in performance level among the skiers investigated in the study of Andersson et al. [13], and that the present study included some of the world’s best XC sprint skiers competing in homogenous heats in world-cup competitions. Extending upon previous findings [14], we found larger correlation coefficients at the last checkpoint in the skating compared to the classical style (τ = ~0.82 vs. ~0.70) for both sexes. This could indicate that competitions in the skating style are more often decided at an earlier stage than competitions in the classical style, which might be due to the difficulty to pass other skiers in the skating style where each skier occupies more of the track width. This was further supported by indications of less accidents in the classical compared to skating style (see Table 1). Moreover, the larger correlation coefficients in the skating style could also explain why there were on average fewer overtaking events in the heats during skating compared to classical competitions (~16 vs. ~20). In the skating style, the highest-ranked skiers more often positioned themselves in the front of the heats at an earlier stage (see Fig 3 and Table 2), and thus controlled the speed in the heat and likely also avoided accidents. Therefore, these findings demonstrate that race tactics might be particularly important in the skating style due to the more limited opportunities to overtake other skiers compared to the classical style.

No significant difference in number of overtaking events between the seasons investigated for both sexes and styles were found, indicating that the number of overtaking events on the same racecourse are relatively consistent. Still, we found a significant sex-difference in the number of overtaking events in one out of the 16 competitions, where women performed more overtaking events than men throughout the racecourse both seasons. This sex-difference was particularly explained by more overtaking events in the middle part of the racecourse. Skiing speed was not examined in this study, but it would have been interesting to know whether speed differences between sexes could explain why women performed more overtaking events in these terrain segments. Furthermore, the competitions analysed had relatively similar conditions, despite some variations (see Table 1). However, differences in external conditions could have influenced the skiers tactical positioning and overtaking events for both sexes, however, this remains to be investigated. Future studies should examine the effectiveness and metabolic cost of the skiers’ race tactics. Altogether, these findings demonstrate that a similar number of overtaking events can be expected in competitions held on the same racecourse under comparable external conditions.

## Conclusions

This study describes the role of tactical positioning in sprint XC skiing. Throughout the heats, there was a gradual increase in the relative importance of being positioned at the front, in which the majority of performance-variance was decided before the start of the finish sprint both among men and women in the classical and skating styles. Notably, the final rank for both sexes was decided at an earlier stage in the skating style compared to the classical style, which is likely explained by greater possibilities for tactical positioning during the heats in the classical style. The number of overtaking events were relatively consistent across seasons, so a similar number of overtaking events can be expected when a competition is repeated on the same racecourse under comparable external conditions. The practical relevance of this study is the novel illustration of how tactical positioning influence performance in sprint XC skiing, and that the number of overtaking events is relatively consistent for competitions performed on the same racecourse.

## Supporting information

S1 Appendix The difference in overtaking events between sexes in the same racecourse over time in two repeated sprint cross-country skiing competitions at four different venues (two classical and two skating style) [(N = 30 male and N = 30 female skiers within each of the eight competitions in this study)]. (PDF)

S1 File Data and analyses conduced in this study. (XLSX)

## References

[1] Andersson E, Supej M, Sandbakk Ø, Sperlich B, Stöggl T, Holmberg HC. Analysis of sprint cross-country skiing using a differential global navigation satellite system. European journal of applied physiology. 2010;110(3):585–95. doi: 10.1007/s00421-010-1535-2 20571822

[2] Tjønnås J, Seeberg TM, Rindal OMH, Haugnes P, Sandbakk Ø. Assessment of Basic Motions and Technique Identification in Classical Cross-Country Skiing. Frontiers in Psychology. 2019;10(1260). doi: 10.3389/fpsyg.2019.01260 31231279 PMC6566644

[3] Stöggl T, Müller E. Kinematic determinants and physiological response of cross-country skiing at maximal speed. Medicine and science in sports and exercise. 2009;41(7):1476–87. doi: 10.1249/MSS.0b013e31819b0516 19516152

[4] Sandbakk Ø, Holmberg HC, Leirdal S, Ettema G. Metabolic rate and gross efficiency at high work rates in world class and national level sprint skiers. European journal of applied physiology. 2010;109(3):473–81. doi: 10.1007/s00421-010-1372-3 20151149

[5] Stöggl T, Müller E, Ainegren M, Holmberg HC. General strength and kinetics: fundamental to sprinting faster in cross country skiing? Scandinavian journal of medicine & science in sports. 2011;21(6):791–803. doi: 10.1111/j.1600-0838.2009.01078.x 20492588

[6] Sandbakk Ø, Holmberg HC, Leirdal S, Ettema G. The physiology of world-class sprint skiers. Scandinavian journal of medicine & science in sports. 2011;21(6):e9–16. doi: 10.1111/j.1600-0838.2010.01117.x 20500558

[7] Losnegard T, Myklebust H, Hallèn J. Anaerobic capacity as a determinant of performance in sprint skiing. Medicine and science in sports and exercise. 2012;44(4):673–81. doi: 10.1249/MSS.0b013e3182388684 21952633

[8] Spencer M, Losnegard T, Hallén J, Hopkins WG. Variability and predictability of performance times of elite cross-country skiers. International journal of sports physiology and performance. 2014;9(1):5–11. doi: 10.1123/ijspp.2012-0382 23799826

[9] Sandbakk Ø, Holmberg HC. A reappraisal of success factors for Olympic cross-country skiing. International journal of sports physiology and performance. 2014;9(1):117–21. doi: 10.1123/ijspp.2013-0373 24088346

[10] Hebert-Losier K, Zinner C, Platt S, Stöggl T, Holmberg HC. Factors that Influence the Performance of Elite Sprint Cross-Country Skiers. Sports medicine. 2017;47(2):319–42. doi: 10.1007/s40279-016-0573-2 27334280 PMC5266777

[11] Sandbakk Ø, Holmberg HC. Physiological capacity and training routines of elite cross-country skiers: Approaching the upper limits of human endurance. International journal of sports physiology and performance. 2017:1–26. doi: 10.1123/ijspp.2016-0749 28095083

[12] Haugnes P, Torvik PØ, Ettema G, Kocbach J, Sandbakk Ø. The effect of maximal speed ability, pacing strategy and technique on the finish-sprint of a sprint cross-country skiing competition. International journal of sports physiology and performance. 2018:1–24. 10.1123/ijspp.2018-0507 30569776

[13] Andersson EP, Govus A, Shannon OM, McGawley K. Sex Differences in Performance and Pacing Strategies During Sprint Skiing. Frontiers in Physiology.2019;10(295). doi: 10.3389/fphys.2019.00295 30967794 PMC6440389

[14] Haugnes P, Kocbach J, Talsnes RK, Noordhof D, Ettema G, Sandbakk Ø. The influence of race tactics for performance in the heats of an international sprint cross-country skiing competition. PloS one. 2022;17(12):e0278552. doi: 10.1371/journal.pone.0278552 36490303 PMC9733856

[15] Seeberg TM, Kocbach J, Wolf H, Talsnes R, Sandbakk Ø. Race development and performance-determining factors in a mass-start cross-country skiing competition. Frontiers in Sports and Active Living. 2023;4:498. 10.3389/fspor.2022.1094254 PMC9871829 36704262

[16] Ainegren M, Linnamo V, Lindinger S. Effects of Aerodynamic Drag and Drafting on Propulsive Force and Oxygen Consumption in Double Poling Cross-country Skiing. Medicine and science in sports and exercise. 2022. doi: 10.1249/MSS.0000000000002885 35142710 PMC9208808

[17] Moxnes JF, Sandbakk Ø, Hausken K. A simulation of cross-country skiing on varying terrain by using a mathematical power balance model. Open access journal of sports medicine. 2013;4:127–39. doi: 10.2147/OAJSM.S39843 24379718 PMC3872006

[18] Moxnes JF, Sandbakk Ø, Hausken K. Using the power balance model to simulate cross-country skiing on varying terrain. Open access journal of sports medicine. 2014;5:89–98. doi: 10.2147/OAJSM.S53503 24891815 PMC4019618

[19] Haugnes P, Kocbach J, Luchsinger H, Ettema G, Sandbakk Ø. The Interval-Based Physiological and Mechanical Demands of Cross-Country Ski Training. International journal of sports physiology and performance. 2019:1–23. doi: 10.1123/ijspp.2018-1007 30958055

[20] McKay AK, Stellingwerff T, Smith ES, Martin DT, Mujika I, Goosey-Tolfrey VL, et al. Defining Training and Performance Caliber: A Participant Classification Framework. International journal of sports physiology and performance. 2022;17(2):317–31. doi: 10.1123/ijspp.2021-0451 34965513

[21] Hopkins WG. A scale of magnitudes for effect statistics. A new view of statistics. 2002;502:411.

[22] McGawley K, Van Waerbeke C, Westberg K-J, Andersson EP. Maximizing recovery time between knock-out races improves sprint cross-country skiing performance. Journal of sport and health science. 2022;11(1):21–9. doi: 10.1016/j.jshs.2021.12.004 34936939 PMC8848028

