# Supplementary material for: The influence of tactical positioning on performance in sprint cross-country skiing
Source: PLoS One. 2023 Jun 23;18(6):e0287717. doi: 10.1371/journal.pone.0287717 (PMC10289379; doi:10.1371/journal.pone.0287717)
Supplement: S1 Appendix — (PDF) [file pone.0287717.s001.pdf]

The differences in overtaking events between groups (sexes) in the same racecourse over time (seasons) in two repeated sprint cross-country skiing competitions at four different venues (two classical and two skating style) for N=30 male and N=30 female skiers within each of the eight competitions in this study:

Interaction effect between sex and seasons on overtakings:

There was no significant interaction between sex and seasons on overtaking events in competition A [(F(1, 14) = 1.21, P = .289, partial η^2 = .080)], competition B [(F(1, 14) = 3.40, P = .086, partial η^2 = .196)], competition C [(F(1, 14) = 1.72, P = .211, partial η^2 = .109)], or competition D [(F(1, 14) = .13, P = .912, partial η^2 = .001)].

The main effect of seasons:

No significant differences in the number of overtaking events were found between seasons for competition A [(F(1, 14) = .73, P = .406, partial η^2 = .050)], competition B [(F(1, 14) = .07, P = .796, partial η^2 = .005)], competition C [(F(1, 14) = 1.45, P = .248, partial η^2 = .094)], or competition D [(F(1, 14) = 4.55, P = .051, partial η^2 = .245)].

The main effect of sex:

There was a significant difference in the number of overtaking events between sexes for competition A [(F(1, 14) = 11.84, P = .004, partial η^2 = .458)], but not for competition B [(F(1, 14) = .04, P = .843, partial η^2 = .003)], competition C [(F(1, 14) = .04, P = .849, partial η^2 = .003)], or competition D [(F(1, 14) = .16, P = .692, partial η^2 = .012)].
